# “Long-term outcomes of ventral hernia repair using heavyweight non-woven polypropylene mesh”

**DOI:** 10.1007/s10029-026-03597-8

**Published:** 2026-02-17

**Authors:** Kayleigh May Risser, Erika M. Schmidt, Kimberly P. Woo, Lucas R. A. Beffa, Ajita S. Prabhu, David M. Krpata, Michael J. Rosen, Clayton C. Petro, Benjamin T. Miller

**Affiliations:** 1 Digestive Disease Institute, Cleveland Clinic Foundation, Cleveland, OH USA; 2 Case Western Reserve University School of Medicine, Cleveland, OH USA; 3 Division of Gastrointestinal Surgery, Northwestern University, Chicago, IL USA

**Keywords:** Nonwoven polypropylene mesh, Heavyweight mesh, Surgimesh WN, Ventral hernia repair

## Abstract

**Purpose:**

Polypropylene mesh is available in the configurations: knitted polypropylene (KP) and nonwoven polypropylene (NWP). Although there is robust literature regarding KP, there is a knowledge gap in the long-term performance of NWP in ventral hernia repair (VHR). We recently reported one-month outcomes of KP versus NWP mesh after VHR; however, long-term outcomes of NWP were not evaluated. This study aims to describe these long-term outcomes.

**Methods:**

Patients who underwent open, clean VHR with Surgimesh WN, a heavyweight NWP mesh, from 2022 to 2025 with one year follow-up were included. Outcomes such as SSO (surgical site occurrence), SSI (surgical site infection), SSOPI (surgical site occurrence requiring procedural intervention), and hernia recurrence were reported at 30-day, one-year, and two-years. Hernia recurrence was defined pragmatically, a composite of patient-reported bulges, clinical exam, and cross-sectional imaging.

**Results:**

160 patients were included. Ventral hernias had a median width of 15 cm. At 30 days, there were 20 SSOs, six SSIs, eight SSOPIs, and no hernia recurrences. At one year, there were three SSOs, one SSI, one SSOPI, and no reoperations for recurrence. At two years (*N* = 35), there was one SSO and no hernia recurrences.

**Conclusion:**

Our early experience with Surgimesh WN indicates it is a safe and effective for VHR. NWP mesh is especially useful given the 50cmx50cm available for large VHRs. Long term follow-up in larger cohorts is needed to support these findings and explore potential advantages of NWP.

**Supplementary Information:**

The online version contains supplementary material available at 10.1007/s10029-026-03597-8.

## Introduction

A recent multicenter clinical trial comparing heavyweight (> 75 g/m^2^) and mediumweight (40–60 g/m^2^) polypropylene mesh in open ventral hernia repairs (VHR) showed comparable patient-reported and clinical outcomes at 30 days and one year postoperatively [1]. Following this study, our group moved towards using heavyweight polypropylene mesh during open, clean VHR in order to avoid central mesh fractures with mediumweight polypropylene mesh, which occurs with an incidence of 4% [2]. Our preferred heavyweight mesh had been knitted polypropylene (KP), but supply chain issues in 2022 limited the availability of our typical heavyweight KP and required us to look for alternatives. As such, we began utilizing a newly available heavyweight non-woven polypropylene (NWP) mesh, Surgimesh WN (BG medical, Deer Park IL).

NWP mesh is created by electrospinning: electrostatic forces produce fibrous scaffolds from biocompatible polymers, like polypropylene. These fibrous scaffolds have a high surface to volume ratio with high porosity and pore interconnectivity [3], properties that have been shown to allow for penetration of macrophages, fibroblasts, collagen, and blood vessels [4]. Mesh size is also a unique feature of Surgimesh WN. The largest size of KP mesh available is 30 cm x 30 cm, but larger mesh sizes are sometimes needed for adequate overlap of the hernia defect. Surgimesh WN is the only commercially available heavyweight polypropylene mesh with sizes as large as 50 cm x 50 cm.

We recently reported 30-day outcomes of NWP mesh in a paper comparing NWP mesh with KP mesh [3], but there is no current long-term report of NWP performance. This study aims to characterize the one-year and two-year outcomes of heavyweight NWP mesh.

## Methods

This is a retrospective review of patients undergoing open VHR with Surgimesh WN at the Cleveland Clinic Foundation between January 2022 and March 2025. Only repairs classified as Centers for Disease Control and Prevention (CDC) wound class I (clean) were included. The Abdominal Core Health Quality Collaborative (ACHQC) was queried for patients eligible for a minimum of one year follow-up. The ACHQC is a nationwide registry which collects health information related to surgical hernia repairs. Data is entered prospectively by participating surgeons and includes patient baseline information, operative details, and follow-up [5]. Institutional Review Board approval was obtained.

Outcomes of interest included wound morbidity at 30 days as well as one and two years postoperatively, including surgical site occurrence (SSO), surgical site infection (SSI), and SSO requiring procedural intervention (SSOPI). SSOs are defined as SSIs, cellulitis, non-healing incisional wounds, fascial disruption, skin or soft tissue ischemia, skin or soft tissue necrosis, serous or purulent wound drainage, stitch abscess, seroma, hematoma, infected or exposed mesh, and/or development of an enterocutaneous fistula [6]. SSIs were grouped into superficial, deep, and organ space infections according to standards set by the CDC [3]. SSOPIs were designated based on whether a patient required wound opening or debridement, suture excision, percutaneous drainage, and/or partial or complete mesh removal.

An additional outcome of interest was hernia recurrence at one and two years postoperatively. Hernia recurrence was defined pragmatically, as a composite of patient-reported outcomes (PRO), clinical exam findings, and radiographic results [7]. Clinical recurrences were any recurrences observed on a clinical exam or on cross-sectional imaging [8, 9]. Patient-reported recurrences, or bulges, may be superseded by clinical exam or radiography, if available [1].

Descriptive statistics were used to summarize demographics and operative characteristics at baseline, 30-day, one-year, and two-year follow-up [10].

## Results

A total of 160 patients were included in the study. Patients had a median age of 61 years, were more likely female (56%) and white (90%), and had a median BMI of 34.3 (Table 1).

**Table 1:** Demographics and Comorbidities

Characteristic	Surgimesh WN [N=160]
Age (years), median [IQR]	61 [53, 70]
Female	89 (56)
BMI (kg/m^2^), median [IQR]	34.3 [30, 38]
Ethnicity	
White	144 (90)
African American	10 (6.2)
Hispanic	0 (0)
Middle Eastern	0 (0)
American Indian or Alaskan Native	0 (0)
Asian, Native Hawaiian, or Other Pacific Islander	1 (0.6)
Other or Unknown	5 (3.1)
Smoker (current within 1 month)	21 (14)
Diabetes Mellitus	58 (36)
Hypertension	119 (74)
COPD	20 (12)
Immunosuppression	13 (8.1)
Anti-platelet Medications	4 (2.5)
History of Abdominal Wall SSI	30 (19)
Prior Prosthetic Mesh Infection	8 (5)
Recurrent Hernia	92 (58)
ASA Class	
1	0 (0)
2	21 (13.1)
3	132 (82.5)
4	7 (4.4)
Number of Previous Repairs	
0	68 (42.5)
1	37 (23.1)
2	30 (18.8)
3	15 (9.4)
4	6 (3.8)
5+	4 (2.5)

Data are presented as number (percentage) of patients [N] unless otherwise indicated

*BMI* body mass index (calculated as weight in kilograms divided by height in meters squared), *COPD* chronic obstructive pulmonary disease, *SSI* surgical site infection, *ASA* American Society of Anesthesiologists

Operative details are summarized in Table 2 and reveal operative times between 60 and 239 min (81.8%) for ventral hernias with median width of 15 cm [IQR 12, 19]. A transversus abdominis release (TAR) was performed in 98% of cases. Anterior fascial closure over retromuscular mesh was achieved in all but two cases.

**Table 2:** Operative details

	Surgimesh WN [N=160]
Myofascial Release	
Transversus Abdominis	156 (98.1)
Mesh Location	
Sublay - retromuscular	158 (99.4)
Hernia Width (cm), median [IQR]	15 [12, 19]
Hernia Length (cm), median [IQR]	23 [18, 27]
Mesh Width (cm), median [IQR]	40 [30, 40]
Mesh Length (cm), median [IQR]	40 [30, 40]
Fascial Closure	158 (98.8)
Mechanical Mesh Fixation Used	12 (7.5)
Concomitant Procedure	6 (3.8)
Time (minutes)	
0-59	1 (0.6)
60-119	32 (20)
120-179	58 (36.2)
180-239	41 (25.6)
≥240	28 (17.5)
Length of Stay (days), median [IQR]	5 [3, 6]

Data are presented as number (percentage) of patients [N] unless otherwise indicated

At 30-day follow-up (*N* = 115), twenty SSOs (17%) occurred, including wound cellulitis (1.7%), wound serous drainage (5.2%), wound purulent drainage (0.9%), seroma (8.7%), and hematoma (2.6%). There was one unspecified SSO (0.9%). There were five superficial SSIs (4.3%) and two deep SSIs (1.7%) reported, as well as eight SSOPIs (7%) (one patient had both superficial and deep SSI). Other notable complications at this time included pulmonary embolism (3.5%), deep vein thrombosis (1.7%), urinary tract infection (3.5%), acute renal failure (2.6%), respiratory failure requiring intubation (1.7%), ventilation for over 48 h (1.7%), post operative bleeding requiring transfusion (4.3%), and pain requiring intervention (3.5%) (Supplemental Table 1). Readmission occurred for four patients (3.5%) due to wound complications (1.7%) and/or gastrointestinal complications (1.7%) (Table 3). Reoperations were performed in two (1.7%) patients for mesh excision and unspecified intra-abdominal pathologies. One death occurred within 44 days of operation. Hernia recurrence did not occur in any patients at 30-day follow-up.

**Table 3:** Outcomes at 30 Days

	Surgimesh WN [N=115]
Wound Morbidity	
SSO	20 (17)
Wound Cellulitis	2 (1.7)
Wound Serous Drainage	6 (5.2)
Wound Purulent Drainage	1 (0.9)
Seroma	10 (8.7)
Hematoma	3 (2.6)
Unspecified	1 (0.9)
SSI	6 (5.2)
Superficial	5 (4.3)
Deep	2 (1.7)
Organ Space	0 (0)
SSOPI	8 (7)
Readmissions	
Wound complications	2 (1.7)
Gastrointestinal complications	2 (1.7)
Reoperation	
Unrelated Intra-abdominal Pathology	1 (0.9)
Mesh Excision	1 (0.9)
Hernia Recurrence	0 (0)
Death Within 44 Days (N=139)	1 (0.7)

Data are presented as number (percentage) of patients [N] unless otherwise indicated

*SSO* surgical site occurrence, *SSI* surgical site infection, *SSOPI *surgical site occurrence requiring procedural intervention

All patients in this study cohort were eligible for one-year follow-up. Of the 160 patients, 121 (76%) had one-year follow-up data available, with six experiencing pragmatic recurrence. There were four (3.6%, *N* = 110) clinical recurrences and five (19%, *N* = 26) patient-reported recurrences. There were no reoperations for hernia recurrence at one year. At one year postoperatively (*N* = 110), wound morbidity was reported as three (2.8%) SSOs, one (0.9%) SSI, and one (0.9%) SSOPI. No mesh excisions occurred. The one-year disease-progression free survival rate is 0.95 (95% CI 0.91 to 0.99) (Table 4).

**Table 4:** Outcomes at 1 Year and 2 Years

	1 Year	2 Years
	[N=110)]	[N=35]
SSO	3 (2.8)	1 (2.9)
SSI	1 (0.9)	0 (0)
SSOPI	1 (0.9)	0 (0)
Hernia Recurrence		
Patient-Reported	5 (19) [N=26]	0 (0) [N=3]
Clinical	4 (3.6)	0 (0) [N=34]
Pragmatic	6 (5) [N=121]	0 (0) [N=37]
Reoperation	1 (1) [N=96]	0 (0) [N=36]
Death	0 (0)	0 (0)

Data are presented as number (percentage) of patients [N] unless otherwise indicated

*SSO* surgical site occurrence, *SSI* surgical site infection, *SSOPI* surgical site occurrence requiring procedural intervention

86 patients were eligible for two-year follow-up, and 37 (43%) had two-year follow-up data available. No pragmatic recurrences or reoperations were observed at two years. At two years postoperatively (*N* = 35), one SSO (2.9%) was reported. The median follow-up duration was 549 days (IQR: 393–699 days).

## Discussion

This study describes the postoperative outcomes at least one year after open, clean retromuscular VHR with NWP heavyweight mesh (Surgimesh WN). In this cohort of patients with large ventral hernias, most of whom underwent VHR with TAR, wound morbidity rates were low and consistent with prior studies of similar patients. There were no mesh infections or mesh excisions reported at one and two years postoperatively. Hernia recurrence rates were also low at one and two years postoperatively. These findings indicate that heavyweight NWP mesh is a suitable option for open, clean retromuscular VHR.

Regarding open, clean retromuscular VHR, heavyweight polypropylene has distinct advantages over mediumweight mesh. Heavyweight polypropylene is more durable than mediumweight polypropylene. Mediumweight polypropylene has a fracture rate approaching 4% in cases with anterior fascial closure and over 30% in cases where the anterior fascia is not closed, or “bridging” scenarios [2]. Compared to mediumweight polypropylene, heavyweight polypropylene has similar outcomes with regard to pain scores and abdominal wall-specific quality of life at one year [1]. Given these factors, heavyweight polypropylene mesh is our preferred prosthetic in open, clean retromuscular VHR. However, while mediumweight KP mesh has been available in a 50 cm x 50 cm size, heavyweight KP mesh was, until recently, only available in a 30 cm x 30 cm size.

One unique advantage heavyweight NWP (Surgimesh WN) holds is its availability in larger sizes than other heavyweight polypropylene mesh. Surgimesh WN is available in 40 cm x 40 cm and 50 cm x 50 cm sizes. Sewing smaller meshes together to create a large “paneled” mesh may be done to repair massive hernias, but it has disadvantages [11]. The meshes must be sewn together with running and interrupted polypropylene suture, and the knots, if they are exposed, may serve as a nidus for developing a suture abscess. Sewing a “paneled” mesh also increases procedure times by up to 45 min [11]. The larger 40 cm x 40 cm and 50 cm x 50 cm heavyweight NWP mesh sizes available eliminate the extra time and resources that were previously needed to create a “paneled” mesh, offering a distinct advantage in the repair of large hernias. Our study did not compare the long-term outcomes of various mesh sizes because cases requiring a larger mesh often have many complexities that make attributing outcomes to mesh size alone inappropriate. Therefore, we chose to evaluate a more holistic group with a wider range of mesh sizes, including 30 cm x 30 cm to 50 cm x 50 cm, to more fairly allow for inevitable comparisons against heavyweight KP mesh.

Although large mesh options are desirable, mesh safety must also be demonstrated and can reasonably be assessed using surrogate parameters such as wound morbidity, readmission, and reoperation. Our study demonstrates acceptable rates of each of these parameters with the use of NWP mesh in clean, retromuscular hernia repairs. In their recent study comparing NWP mesh to KP mesh, Fafaj et al. identified no significant differences in readmission, reoperation, or hernia recurrence between groups [3]. Notably, NWP mesh was associated with a greater incidence of deep SSI, however, both clean and contaminated cases were included in contrast to our study [3]. We demonstrated comparable rates of SSO (17%) and SSI (5.2%) at 30 days with Surgimesh WN to that previously reported for heavyweight mesh by Krpata et al. (12% and 4.8% respectively) [1]. Wound complications, recurrences, and reoperations were similarly found to be rare events at one and two years in our study. Together, the consistency of favorable short-term outcomes reported for NWP mesh taken with the overall low rate of long-term adverse clinical outcomes found in this study indicate that heavyweight NWP mesh has an acceptable safety profile compared to alternative heavyweight mesh options utilized for open, retromuscular VHRs.

Mesh efficacy must also be evaluated, which we assessed using hernia recurrence rates. One-year clinical and pragmatic hernia recurrence rates for NWP mesh (Surgimesh WN) were 3.6% and 5%, respectively, which are similar to recurrence rates from prior studies of retromuscular VHRs using heavyweight mesh. At one year postoperatively, Krpata et al. reported clinical hernia recurrence rates and pragmatic hernia recurrence rates of 1.2% and 8.1%, respectively, in patients undergoing VHR with TAR and implantation of heavyweight KP mesh [1]. Additionally, a 2023 meta-analysis of open, retromuscular VHRs by Trinidade et al. revealed hernia recurrence rates ranging from 0% to 9.76% for various studies analyzing heavyweight mesh outcomes at one year [12]. In the context of open, retromuscular VHRs, heavyweight NWP mesh appears to perform similarly to other heavyweight meshes with respect to hernia recurrences.

This study is not without limitations. This is a retrospective, registry-based analysis of the ACHQC which depends on surgeon-reported outcomes for data collection, excluding any cases from physicians who do not participate in the reporting procedure. As such, the data reported here may be subject to biases and/or inaccurate or missing variables. Given that not every patient operated on by our group during the time period of this study received heavyweight NWP mesh, our findings may also be influenced by selection bias. Further, we acknowledge these results represent that of a high-volume hernia center which may limit their generalizability. We also recognize that two-year follow-up was available for only 35 of 160 patients, and more robust two-year outcome data are needed. As it is possible that two-year follow-up is not sufficient to determine the long-term outcomes of VHR with heavyweight NWP mesh, additional studies with larger cohorts are required to obtain more robust follow-up and further clarify trends in wound morbidity and hernia recurrence both at two years and beyond, particularly given the limited availability of two-year follow-up in this study. Future prospective studies will also enable examination of patient-reported outcomes, including postoperative hernia-specific quality of life and pain intensity, which are essential in continuing to evaluate both the short- and long-term performance of Surgimesh WN. Cost data regarding Surgimesh WN, specifically regarding the larger 50 cm x 50 cm device, was unavailable for this study and should be considered as an important factor for future investigation.

## Conclusion

Our early experience with Surgimesh WN, including the one- and two-year recurrence and wound morbidity outcomes reported in this study, indicates it is a safe and effective option for open retromuscular VHR. NWP mesh is especially useful given the availability of the 50 cm x 50 cm size for large hernia repairs. Further long-term follow-up in larger patient cohorts are needed to support these findings and explore potential advantages of NWP mesh for open retromuscular VHR.

## Supplementary Information

Below is the link to the electronic supplementary material.

Supplementary Material 1 (DOCX 21.5 KB)

## Data Availability

The ACHQC data is available to ACHQC statisticians who provided the analysis. Requests to access datasets should be directed to the website: https://.achqc.org.
