# Supplementary material for: “Long-term outcomes of ventral hernia repair using heavyweight non-woven polypropylene mesh”
Source: Hernia. 2026 Feb 17;30(1):95. doi: 10.1007/s10029-026-03597-8 (PMC12913288; doi:10.1007/s10029-026-03597-8)
Supplement: Supplementary file 1 — Supplementary Material 1 (DOCX 21.5 KB) [file 10029_2026_3597_MOESM1_ESM.docx]

Supplemental Table 1. Non-Hernia Related Postoperative Complications

_____________________________________________________________________________________________

**Complication Type** **30 Days**

[N=115]

_____________________________________________________________________________________________

Pulmonary Embolism 4 (3.5)

Stroke 0 (0)

DVT 2 (1.7)

Sepsis 1 (0.9)

Septic Shock 1 (0.9)

MI 0 (0)

Cardiac Arrest 1 (0.9)

UTI 4 (3.5)

Renal Insufficiency 0 (0)

Acute Renal Failure 3 (2.6)

Pneumonia 3 (2.6)

Respiratory Failure Requiring Intubation 2 (1.7)

Ventilator > 48 hours 2 (1.7)

Coma > 24 hours 1 (0.9)

Peripheral Nerve Injury 0 (0)

Postoperative Bleeding Transfusion 5 (4.3)

Graft/Prothesis/Flap Failure 0 (0)

Pain Requiring Intervention 4 (3.5)

Other 4 (3.5)

Data are presented as number (percentage) of patients [N] unless otherwise indicated. Abbreviations: DVT, deep vein thrombosis; MI, myocardial infarction; UTI, urinary tract infection
